# Uncovering the impact of outliers on clusters’ evolution in temporal data-sets: an empirical analysis

**DOI:** 10.1038/s41598-024-75928-7

**Published:** 2024-12-28

**Authors:** Muhammad Atif, Muhammad Farooq, Muhammad Shafiq, Tmader Alballa, Somayah Abdualziz Alhabeeb, Hamiden Abd El-Wahed Khalifa

**Affiliations:** 1 Department of Statistics, University of Peshawar, Peshawar, Pakistan; 2 Institute of Numerical Sciences, Kohat University of Science and Technology, Kohat, Pakistan; 3 Department of Mathematics, College of Science, Princess Nourah bint Abdulrahman University, P.O. Box84428, Riyadh, 11671 Saudi Arabia; 4 Department of Mathematics, College of Science, Qassim University, Buraydah, 51452, Saudi Arabia; 5 Department of Operations and Management Research, Faculty of Graduate Studies for Statistical Research, Cairo University, Giza, 12613 Egypt

**Keywords:** Streaming data, Clustering, Outliers, Change detection, Statistical model, Stochastic systems, Transition, Mathematics and computing, Applied mathematics, Statistics

## Abstract

This study investigates the impact of outliers on the evolution of clusters in temporal data-sets. Monitoring and tracing cluster transitions of temporal data sets allow us to observe how clusters evolve and change over time. By tracking the movement of data points between clusters, we can gain insights into the underlying patterns, trends, and dynamics of the data. This understanding is essential for making informed decisions and drawing meaningful conclusions from the clustering results. Cluster evolution refers to the changes that occur in the clustering results over time due to the arrival of new data points. The changes in cluster solutions are classified as external and internal transitions. The study employs the survival ratio and history cost function to investigate the effects of outliers on changes experienced by the clusters at successive time points. The results demonstrate that outliers have a significant impact on cluster evolution, and appropriate outlier handling techniques are necessary to obtain reliable clustering results. The findings of this study provide useful insights for practitioners and researchers in the field of stream clustering and can help guide the development of more robust and accurate stream clustering algorithms.

## Introduction

Modern applications often involve dynamic data streams that originate from various sources. Streaming data is becoming increasingly critical in numerous applications, including network intrusion detection, transaction streams, phone records, internet click-streams, social streams, and weather monitoring. As a result, researchers have been focusing on analyzing streaming data, exploring ways to effectively store, query, analyze, extract, and predict important information from data streams. Ongoing research is dedicated to identifying the best practices for managing evolving data streams^1,2^.

To extract knowledge from massive volumes of continually generated streams, data stream mining is an active research area. In this context, several data stream algorithms have been proposed to perform unsupervised learning^3^. One of these models in data mining study is the clustering approach. Streaming data arrives in fragments $X_1,\ X_2, \ldots , X_n$, where each $X_i$ is a collection of points that can be clustered in the main memory^4^. Clustering is the process of categorizing a set of diverse data objects into meaningful groups, based on their degree of similarity or proximity. Once the clusters are identified, each group is given a specific label or tag for easy identification^5^. However, unlike the batch clustering algorithms, stream clusters are not static. Rather the cluster solutions evolve over time due to non-stationary continual generation of streaming data. Several models and algorithms have been introduced in the literature for monitoring changes in clustering solutions of streaming and temporal datasets. However, It should be noted that cluster analysis may not be suitable for analyzing all types of data; in fact, some types of data behaviour might significantly skew the results. One of these tendencies is brought on by the existence of outliers, which are described as anomalous numbers with the potential to significantly affect the study^6,7^. Properly identifying these outliers and calculating their actual impact on data behaviour are not always straightforward tasks^8^. As a result, scholars often follow recommended practices that propose excluding these outliers, treating them as a hurdle that must be cleared before delving into the primary analysis^9,10^.

The purpose of this paper is to examine the impact of outliers on the evolution of clustering solutions in streaming datasets. To our knowledge, so far, no study is conducted that analyze the impact of outliers on the clustering of temporal datasets and the transitions adopted by the clusters as a result of new data items. Due to the lack of literature and no guidance available in such complex situation we analyze the synthetic data along with the real-life examples and examine the cluster’s evolution.

## Background

In this section, an overview of the technical issues is covered along with a review of the literature on contemporary streaming clustering techniques. This section also provides a summary of the work’s motivating factors and contributions.

### A review of state-of-art methods

In recent years, a number of techniques for clustering streaming data have been developed as shown in^3,11–16^. As one of the earliest algorithms, Chakrabarti et al.^17^ proposes the evolutionary clustering algorithm for clustering temporal datasets. This model generates a series of clustering solutions $\left\{ \xi _1, \xi _2, \ldots , \xi _n \right\}$ at corresponding time points $\left\{ t_1, t_2, \ldots , t_n \right\}$ from temporal datasets. Where $\xi _i = \left\{ C_1(t_i), C_2(t_i), \ldots , C_{k_i}(t_i) \right\}$ is the set of clusters obtained at time-stamp $t_i$. The quality of the clustering sequence is then computed using the mathematical expression:

1 $$\begin{aligned} \sum _{t=1}^{T} {\texttt {sq}}(\xi _t,\ M_t) - {\texttt {cp}} \sum _{t=2}^{t} {{\texttt {hc}}}(\xi _{t-1}, \ \xi _t) \end{aligned}$$

where ${\texttt {sq}}(\xi _t,\ M_t)$ is the snapshot quality that returns the quality of clustering $\xi _t$ with respect to proximity $M_t.$ Similarly, ${{\texttt {hc}}}(\xi _{t-1},\ \xi _t)$ is the history cost ${{\texttt {hc}}}$ function that compute the quality of clustering $\xi _t$ at time point *t*. This is a metric used to evaluate the performance and stability of clustering algorithms over multiple iterations or time points in dynamic data environments. It measures the consistency and quality of clustering solutions by comparing the current clustering solution with the solutions from previous iterations or time points. The ${{\texttt {hc}}}$ function quantifies the degree of similarity between successive clustering solutions. A low ${{\texttt {hc}}}$ indicates that the current clustering solution is similar to previous solutions, suggesting stability and consistency in the clustering process. Conversely, a high value implies significant differences between the current and previous solutions, indicating instability or inconsistency in the clustering results. The $cp > 0$ is a user-defined parameter that trade-off between these two functions. The *k*-means and hierarchical clustering techniques are taken into consideration using this framework. This framework is extended to spectral clustering^18^, density-based clustering^19^, and Hierarchical Dirichlet Process with the Hidden Markov model^20^.

Denny and Squire^21^ proposed a technique that leverages self-organizing maps to identify changes in the cluster solutions of temporal data across two time periods. This approach was based on comparing the clustering results at various time intervals and tracking changes between the current and prior results. The algorithm is capable of tracing splitting and merging clusters at subsequent time points. However, this technique neglects the identification of the newly emerging and disappearing clusters. This problem was overcome by the Relative Density Self-Organizing Map (ReDSOM) introduced by Denny et al.^22^, a visualization-based algorithm that compares clustering solutions of temporal datasets.

Spiliopoulou et al.^23^ introduces the MONIC framework employed to model and track changes in clustering solutions of cumulative data streams. This framework compares the clustering solutions obtained at two successive time points and traces the changes in the new solution regarding the previous one. The changes adopted by the clusters are broadly classified into two categories, i.e. external and internal transitions. External transitions of clusters refer to changes that occur in the composition of clusters over time, particularly in response to the arrival of new data points. These changes involve the emergence, disappearance, splitting, merging, or survival of clusters. For example, in a scenario where new data points introduce a distinct pattern or trend, existing clusters may need to adjust their boundaries or merge with other clusters to better capture the evolving structure of the data. Conversely, if certain clusters become less representative of the data distribution or are no longer relevant, they may dissolve or split into smaller clusters. On the other hand, the internal transition includes changes in size, cohesion, and location of the survived candidates. These changes may occur due to shifts in the distribution of data points within the cluster, changes in the centroid or mean of the cluster, or fluctuations in the density or spread of data points. The notion of the MONIC framework is based on a non-symmetric matrix described as overlap and is represented by the following expression:

2 $$\begin{aligned} {\texttt {Overlap}}(C_a(t_i), \ C_b(t_j)) = \frac{\left| C_a(t_i) \bigcap C_b(t_j) \right| }{\left| C_a(t_i) \right| } \, a = 1,2,\ldots ,\ k_1 \, \ b = 1,2,\ldots \, k_2 \end{aligned}$$

where $C_a(t_i)$ belongs to the set of clusters obtained at first clustering and $C_b(t_j)$ belongs to the set of clusters obtained at second clustering. This generates a matrix of order $k_1*k_2$, where $k_1$ and $k_2$ is the number of clusters from first and second clustering respectively. The value on the matrix’s corresponding element is the similarity index between clusters and serves as an indicator for tracing the external transition. In contrast, cluster membership is assessed to trace the internal transition of the surviving clusters. The Monitoring Clusters Transition (MClusT) algorithm introduced by Oliveira and Gama^24^ visualizes the clusters’ transition on a bipartite graph using the conditional probabilities as the edge weights.

3 $$\begin{aligned} {\texttt {weight}}(C_a(t_i), \ C_b(t_j))= & P\left( X\epsilon C_b\left( t_j \right) \mid X\epsilon C_a\left( t_i \right) \right) \end{aligned}$$

4 $$\begin{aligned}= & \frac{\sum P\left\{ X\epsilon \left( C_a\left( t_i \right) \bigcap C_b\left( t_j \right) \right) \right\} }{\sum P\left\{ X \epsilon C_a\left( t_i \right) \right\} } \end{aligned}$$

MClusT includes a taxonomy of transitions, a tracking method based on Graph Theory, and a transition detection algorithm. The conditional probabilities are measured for every possible pair of clusters obtained at consecutive time points in the stream. These conditional probabilities serve as an indicator for monitoring the cluster solutions.

Monitoring and tracking changes in clusters of temporal data is essential in various domains, including finance, healthcare, and security, where real-time decision making is critical. The evolution of clusters over time can provide valuable insights into the underlying patterns and trends in the data, which can inform decision-making processes. For instance, in financial trading, monitoring the evolution of stock clusters can help traders identify potential investment opportunities and mitigate risks. In healthcare, monitoring the evolution of disease clusters can help medical practitioners identify emerging epidemics and devise appropriate intervention strategies. Atif et al.^25^ shows the significance and applications of monitoring and tracking changes in clusters using various real-life data-sets.

### Problem statement

The problem addressed in this article is the impact of outliers on cluster evolution in temporal data-sets. Clustering is a fundamental technique for exploratory data analysis, which aims to group data points into meaningful clusters based on their similarity. However, clustering temporal data-sets requires methods that can capture the temporal dependencies between data points, and the presence of outliers can significantly impact the clustering structure and affect the clustering results. Outliers are data points that deviate significantly from the typical pattern of the data, and their presence can distort the clustering structure and affect the evolution of clusters over time. Although there have been significant efforts in the literature to develop methods for stream clustering and outlier detection, there is a lack of research on the impact of outliers on cluster evolution in temporal data-sets. Therefore, the problem addressed in this article is to investigate how outliers affect the changes experiences by clusters at successive time points in temporal data-sets. The study explores both external and internal transitions of cluster solutions. We aim to quantify the influence of outliers on cluster evolution and provide an empirical analysis of their impact using the survival ratio (${\texttt {SR}}$) and ${{\texttt {hc}}}$ function. The ${\texttt {SR}}$ is a metric used to assess the persistence of clusters across successive time points in dynamic data environments. It measures the proportion of clusters that maintain their existence or survival from one time point to the next. Our findings will have important implications for stream clustering and anomaly detection in temporal data-sets^26^.

### Challenges, motivation and contributions

This article faces several challenges related to the analysis of outlier impact on cluster evolution in temporal data-sets. One of the major challenges is the selection of appropriate metrics to quantify the influence of outliers on cluster evolution accurately. Usually unsupervised learning problems are challenging, especially in case of streaming data, where the cases are abundant but without true class labels. Because true labels are unavailable so changes in the clusters are hard to identify. In such a complex situation the simulation of streams and performance indicators were hard to identify. As stated earlier the presence of outliers may influence ${{\texttt {hc}}}$ function of clustering solutions. As a result very inconsistent clusters with historic datasets are obtained at successive time points. These challenges inspired us to compose this study, in which we examine the effects of outliers on the evolution of the cluster at discrete points in temporal datasets. To address these challenges, this article proposes an empirical analysis using the ${\texttt {SR}}$ and ${{\texttt {hc}}}$ function to quantify the impact of outliers on cluster evolution in simulated and real-life temporal data-sets.

### Data streams

In the study, both synthetic and real-life streams were utilized to generate and validate the results. The synthetic streams were created with predetermined characteristics at two discrete time points, referred to as time point $t_1$ and time point $t_2$. These synthetic streams allowed the researchers to control and manipulate the data to study the impact of outliers on cluster evolution under different scenarios. In the simulation of the data-sets, several parameters were considered to ensure the generation of diverse and representative data. These parameters encompassed the number of features or variables present in the dataset, the degree of separation between neighboring clusters, the covariance structure between variables, the number of true classes within the data, the number of clusters generated through clustering algorithms, and the presence of outliers. Each of these parameters played a crucial role in shaping the characteristics of the simulated datasets, thereby enabling a comprehensive evaluation of the impact of outliers on cluster evolution over time. By carefully controlling these parameters, we aimed to create datasets that accurately reflected the complexities and dynamics observed in real-world data scenarios. Additionally, real-life streams were incorporated into the study, providing actual data from real-world datasets. These real-life streams captured the complexity and diversity present in practical applications and enabled the assessment of the proposed outlier handling techniques on real-world data. Table 1 below represents synthetic and real-life streams that were used to generate and validate results of this study. All synthetic streams were simulated at two discrete points in time.

**Table 1:** Data streams used for generating results of the study.

Data-sets	Instances	Features	Separation	Clusters	Predicted clusters	Outliers	% Outliers
$D_1$ (Synthetic)	24,000	2	− 0.1	3	3	No	–
$D_2$ (Synthetic)	24,000	2	− 0.1	3	3	Yes	10
$D_3$ (Synthetic)	24,000	2	− 0.1	3	3	Yes	20
$D_4$ (Synthetic)	24,000	2	− 0.1	3	3	Yes	30
$D_5$ (Synthetic)	24,000	2	0.0	3	3	No	–
$D_6$ (Synthetic)	24,000	2	0.0	3	3	Yes	10
$D_7$ (Synthetic)	24,000	2	0.0	3	3	Yes	20
$D_8$ (Synthetic)	24,000	2	0.0	3	3	Yes	30
$D_9$ (Synthetic)	24,000	2	0.1	3	3	No	–
$D_{10}$ (Synthetic)	24,000	2	0.1	3	3	Yes	10
$D_{11}$ (Synthetic)	24,000	2	0.1	3	3	Yes	20
$D_{12}$ (Synthetic)	24,000	2	0.1	3	3	Yes	30
$D_{13}$ (Real)	–	–	–	–	–	–	

All the synthetic streams were simulated at two time points. These streams were generated using covariance values of 0, 5, and 10 between the variables

## Methods

This section provide a detailed discussion on the algorithms and techniques employed for outlier detection, cluster analysis, and data visualization. Additionally, we describe the statistical methods used for data analysis and interpretation.

### Cluster analysis

The *k*-means, *k*-modes, and Hierarchical clustering algorithms were implemented to the datasets for generating the cluster solutions. The true number of classes (i.e. number of clusters generated in respective simulation) was used as a relevant value of *k* in the streams. The usual Euclidean distance function was used as a dis-similarity measures between objects for generating the clustering solutions.

### Outlier detection techniques

Outliers significantly impact the stability and accuracy of clustering algorithms. Therefore, robust outlier detection techniques are essential for ensuring the reliability of cluster analysis. Here are some popular distance-based outlier detection techniques:

Distance-based is the most commonly used outlier detection technique. This detection techniques rely on measuring the distance between data points to identify outliers. These methods assume that outliers are typically located far away from the majority of the data points or exhibit unusually large distances from their nearest neighbors. Common distance metrics used in these methods include Euclidean distance, Mahalanobis distance, and Manhattan distance.

Statistical techniques such as modified Z-scores, Grubbs’ test, Dixson’s Q test, and interquartile range (IQR) can be used to detect outliers in the dataset. Statistical outlier detection methods are based on well-established statistical principles. These techniques focus on identifying data points that deviate significantly from the expected statistical properties of the dataset. These methods typically involve statistical models or hypothesis testing to detect observations that are unlikely to occur under a given distribution.

Clustering-based outlier detection techniques, on the other hand, identify outliers by leveraging the clustering structure of the data. These methods partition the dataset into clusters and then identify observations that are distant from their respective cluster centroids or have low cluster membership probabilities. Similarly, density-based Methods identify outliers based on the density of data points within a given region. Outliers are often defined as points with significantly lower density compared to their neighbors.

In our study, outliers are introduced into simulated datasets by strategically placing them at a distance from the cluster centroids. These outliers are defined as data points that deviate significantly from the typical behavior of the majority of observations in the dataset. Specifically, outliers are observations that lie at an abnormal distance from the center of their respective clusters or exhibit unusual patterns compared to the rest of the data. By simulating outliers in this manner, we aim to mimic real-world scenarios where anomalous observations exist in the data, challenging the clustering algorithms to accurately identify and differentiate them from the regular data points. This approach allows us to systematically evaluate the performance of outlier detection techniques and their impact on the evolution of clusters over time in streaming datasets.

### Generalized additive models

Generalized Additive Models (GAMs) offer a flexible framework for modeling complex relationships between predictor variables and a response variable, allowing for nonlinear and nonparametric relationships to be captured effectively. Unlike traditional linear models, GAMs do not impose linearity assumptions on the predictor-response relationship, making them suitable for analyzing data with nonlinear or non-monotonic patterns. In GAMs, each predictor variable is allowed to have a smooth, nonlinear effect on the response variable, represented by smooth functions. These functions are estimated from the data, allowing for the identification of intricate relationships that may not be captured by linear models. Let *Y* denote the response variable, and $X_1, X_2,\ldots , X_p$ denote the predictor variables. Then, the GAM model can be expressed as follows:

5 $$\begin{aligned} Y = f_1(X_1) + f_2(X_2) + \cdots + f_p(X_p) + \epsilon \end{aligned}$$

where $f_1(X_1), f_2(X_2),\ldots , f_p(X_p)$ are smooth functions of the predictor variables and $\epsilon$ represents the error term. GAMs are particularly useful in situations where the relationship between predictors and the response is unknown or difficult to specify a priori. By incorporating smooth functions, GAMs provide a flexible and interpretable approach to modeling complex data, making them a valuable tool in data analysis and predictive modeling.

In this study, we employ the history ${{\texttt {hc}}}$ as the response variable. The predictor variables include the percentage of outliers in the dataset, the covariance structure, and the separation between neighboring clusters. These predictor variables are utilized to examine their impact on the behavior of the clustering algorithm and the evolution of clusters over time.

### Methodology

Consistency of clusters over time is an important aspect of cluster analysis in temporal data-sets. The evolution of clusters over time can be influenced by various factors, including changes in the data distribution, emergence of new patterns, or the presence of outliers. Therefore, it is crucial to evaluate the consistency of cluster solutions over time and identify any significant changes or discrepancies in the clustering results. Atif and Leisch^27^ developed an R package *clusTransition* for monitoring and tracking changes in cluster solutions of temporal data-sets. In this research article, we investigate the consistency of cluster solutions over time and the impact of outliers on the evolution of these clusters. We utilize the *clusTransition* package to monitor and track the changes in cluster solutions of temporal data-sets and evaluate the consistency of the clustering results using various stability indices. By analyzing the consistency of cluster solutions over time, we can better understand the stability and reliability of the clustering results and identify any potential issues or challenges that may arise due to the presence of outliers.

The ${{\texttt {hc}}}$ function is a useful measure for evaluating the consistency of cluster solutions over time, as it incorporates both the spatial proximity and temporal continuity of the clusters. By penalizing significant deviations from previous time points, the ${{\texttt {hc}}}$ function can identify instances where the underlying data distribution has changed significantly, leading to the emergence or dissolution of clusters. In this research article, we use the ${{\texttt {hc}}}$ function to assess the impact of outliers on the consistency of clusters over time and to evaluate the performance of our proposed method for handling outliers in temporal data-sets. In simple words the cluster solution should not deviate drastically from the clustering solution at previous time stamp. We used the ${{\texttt {hc}}}$ function as a performance measure i.e. the impact of outliers is investigated on the ${{\texttt {hc}}}$ function. The ${{\texttt {hc}}}$ is computed using equation given by:

6 $$\begin{aligned} hc = \left\{ \sum _{i=1}^{n}w_{i}\left( C_{a}^{\prime}\left( t_i \right) - C_{b}^{\prime}\left( t_j \right) \right) \right\} ^{\frac{1}{2}} \end{aligned}$$

where $C_{a}^{\prime}\left( t_i \right)$ is the centroids of the clusters obtained at first clustering, $C_{b}^{\prime}\left( t_j \right)$ is the centroid of the clusters obtained at second clustering, and $w_i$ is the weights assigned to these clusters and represents the cluster cohesion.

Similarly, as the cluster at successive time points are simulated using same cluster centroids. This means that the clusters at time stamp $t_2$ should survive. So, ideally the clusters should survive from time point $t_1$ to time point $t_2$ and no transition should be detected. Hence, we should obtain a high survival ratio, which can be computed using equation given by:

7 $$\begin{aligned} SR = \frac{\left| C_a\left( t_i \right) \epsilon \xi _i \mid C_b\left( t_j \right) \epsilon \xi _j \right| }{k_i} \end{aligned}$$

The generalized additive models was fitted to the data using ${{\texttt {hc}}}$ function as response variable and proportion of outliers and covariance structure as explanatory variables. The model can be represented from the mathematical expression given by:

8 $$\begin{aligned} g\left( E\left( hc \right) \right) = \alpha + s\left( x_1 \right) + s\left( x_2 \right) \end{aligned}$$

where ${{\texttt {hc}}}$ is the dependent variable and ${\texttt {g}}$ is the link function that links the expected value of the dependent variable to the predictors $x_1$ (proportion of outliers) and $x_2$ (covariance structure). The term ${\texttt {s}}$ is the non-parametric smoothing function, which means that the data solely determine the shape of the predictor function^28^.

## Results

Supervised learning is evaluated by comparing the predicted class with the actual class labels of the resulting characteristic. However, in unsupervised learning, actual class labels are not provided to the learning algorithms, making it challenging to assess their effectiveness^29^. To overcome this challenge, simulated datasets were used to achieve the study objectives, and streams were generated for covariance values of 1, 5, and 10 between variables. Due to a lack of relevant literature, identifying performance indicators in this research study posed a challenge. The study employed the survival ratio as a performance indicator to assess the impact of outliers on cluster transitions, as it is a measure of stability.

**Fig. 1:**
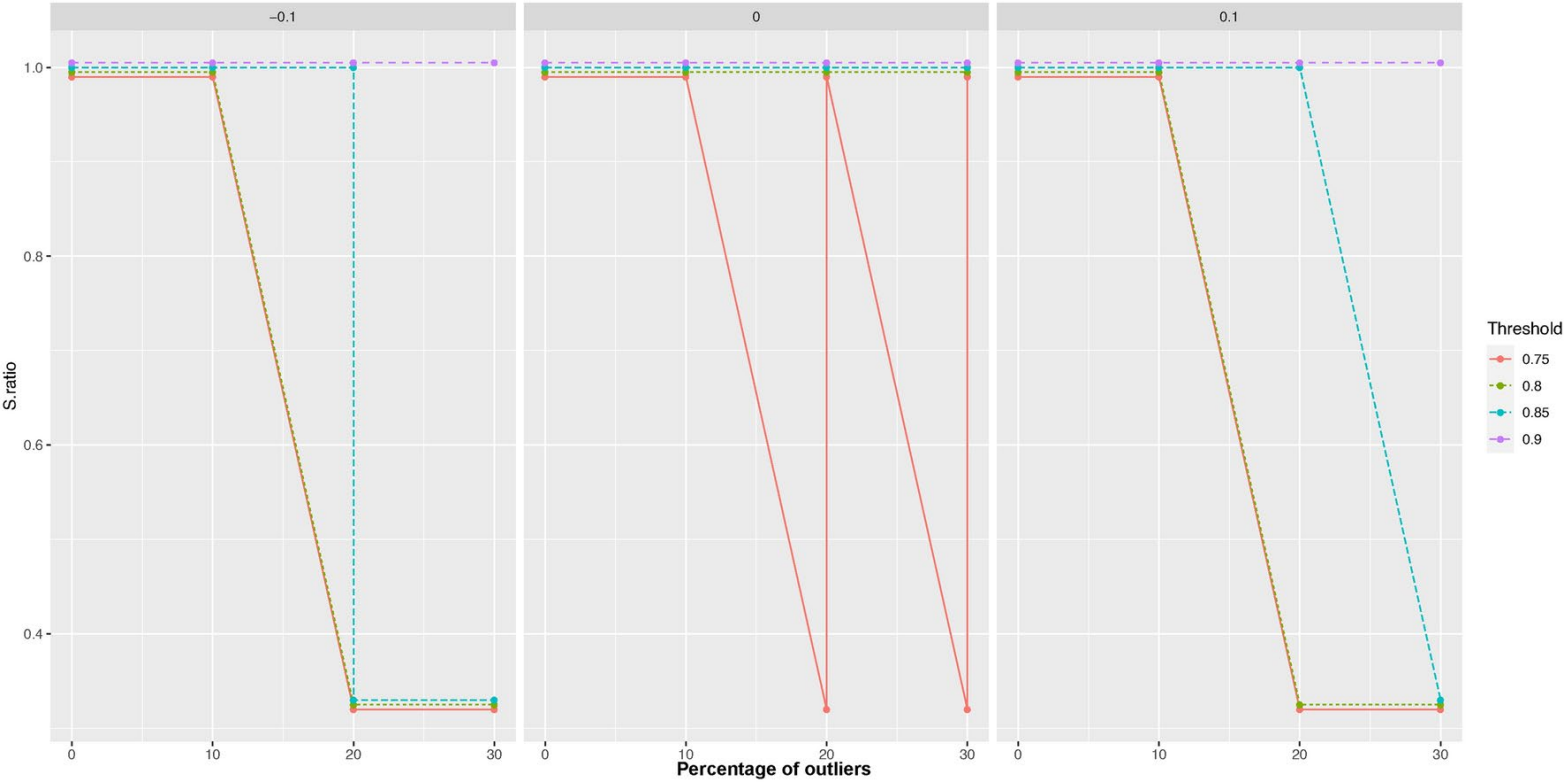
Clusters undergoing external transition in relation to the percentage of outliers for various separation values. The panels represents the separation between neighboring clusters.

Figure 1 illustrates the impact of the survival threshold parameter on the external transitions of the clusters at successive time points for different levels of separation between neighboring clusters. The x-axis represents the percentage of outliers in the data set, while the lines in the figure indicate the user-defined survival threshold parameter. As shown in the figure, when the survival threshold parameter is set to values smaller than or equal to 0.8, significant changes in the clusters can be observed, consistent with previous findings^23^. However, increasing the survival threshold parameter beyond 0.8 produces more stable clustering solutions that remain consistent with the data from earlier time periods. This suggests that the clustering solutions are resilient and not influenced by outliers in the data stream. Nevertheless, as noted in previous studies, even small values of the survival threshold parameter can result in changes in the clusters if there is a noticeable grouping structure. Therefore, careful selection of the survival threshold parameter is essential for ensuring the consistency of the clusters over time.

Given the significance of the separation between neighboring clusters in cluster evolution, this study focuses on investigating the internal transitions for different separation values. The separation between clusters refers to the distance or dissimilarity between the centroids or representative points of adjacent clusters. By analyzing the internal transitions at varying separation values, we can gain insights into how the clustering structure and cohesion change as clusters evolve over time. The study explores the effects of different separation values on the internal transitions of clusters. This involves examining how the cohesion and dispersion of clusters are influenced by varying degrees of separation. By evaluating the changes in cluster characteristics, such as compactness, density, and connectivity, we can understand how different separation values impact the stability and quality of the clustering results.

Table 2 illustrates the calculated ${{\texttt {hc}}}$ values during the second clustering, employing different algorithms with a separation value (${\texttt {Sep}}$) set at − 0.1. The numerical values in the table represent the computed ${{\texttt {hc}}}$ values for each combination of the clustering algorithm and the outlier percentage. These values serve as indicators of how much the clustering solutions deviate or change. Higher ${\texttt {hc}}$ values signify more significant differences between the current clustering result and previous iterations, thus reflecting the stability and consistency of the clustering solutions. The table offers a comprehensive insight into the behavior of different clustering algorithms across varying outlier scenarios. It facilitates the evaluation of clustering algorithm performance for tasks involving covariance structure, assisting in the selection of suitable algorithms based on their stability and adaptation to different levels of outliers. As the percentage of outliers increases, the values of the ${\texttt {hc}}$ metric also tend to rise. Notably, partitioning algorithms exhibit improved performance in the presence of outliers within the data-set, particularly in relation to the cost function. Interestingly, the *k*-modes algorithm displays relatively minimal sensitivity to outliers when clustering temporal data-sets. Moreover, the covariance structure among variables also contributes to shaping the performance of clustering algorithms, influencing their ability to accurately trace the development of clusters over time.

**Table 2:** The history cost function for Sep = − 0.1.

Covariance structure	Clustering algorithm	Percentage outlier			
		0%	10%	20%	30%
1	*k*-means	0.009848	0.40539	0.749778	1.10957
	*k*-modes	0.001803	0.35135	0.617113	1.03931
	Hierarchical	0.064889	0.59413	0.874809	1.21702
5	*k*-means	0.043138	0.59923	1.120076	1.77752
	*k*-modes	0.009355	0.43520	1.010960	1.33251
	Hierarchical	0.043138	0.59923	1.120076	1.97223
10	*k*-means	0.105241	0.76141	1.199975	2.35558
	*k*-modes	0.053209	0.44600	1.109007	2.06382
	Hierarchical	0.314110	0.93113	2.001995	2.80302

Tables 3 and 4 illustrates the calculated ${\texttt {hc}}$ values during the second clustering, employing different algorithms with a separation value ($\texttt {Sep}$) set at 0.0 and 0.1 respectively. Increasing the separation between neighboring clusters did not improves the cost function at successive time points.

**Table 3:** The history cost function for Sep = 0.0.

Covariance Structure	Clustering Algorithm	Percentage Outlier			
		0%	10%	20%	30%
1	*k*-means	0.009848	0.406339	0.753031	1.118398
	*k*-modes	0.001152	0.243116	0.531101	1.018011
	Hierarchical	0.087455	0.599012	0.791879	1.581890
5	*k*-means	0.021472	0.616043	1.129739	1.707061
	*k*-modes	0.010289	0.299323	1.078135	1.580013
	Hierarchical	0.202001	0.818700	1.262991	1.881010
10	*k*-means	0.054572	0.762432	1.401809	2.163485
	*k*-modes	0.011496	0.475380	1.259730	2.096893
	Hierarchical	0.235972	0.875691	1.635974	2.857663

**Table 4:** The history cost function for Sep = 0.1.

Covariance Structure	Clustering Algorithm	Percentage Outlier			
		0%	10%	20%	30%
1	*k*-means	0.009848	0.404816	0.77219	1.105236
	*k*-modes	0.009815	0.201752	0.47201	0.512693
	Hierarchical	0.017893	0.462413	0.87110	1.209134
5	*k*-means	0.022492	0.616478	1.12365	1.723328
	*k*-modes	0.001532	0.338619	1.01522	1.297188
	Hierarchical	0.135296	0.966807	1.62001	2.117320
10	*k*-means	0.053893	0.751432	1.39749	2.253541
	*k*-modes	0.009331	0.504039	1.18962	1.993914
	Hierarchical	0.334981	1.700214	1.89900	2.821454

The history cost function ${\texttt {hc}}$ is computed using equation (3.1)

The hard clustering algorithm was used to generate the clusters from simulated datasets. The hard clustering algorithms divide a dataset into distinct, non-overlapping clusters, where each data point belongs to exactly one cluster. The absorption of new data items containing outliers by the initial clusters resulted in the detection of no external transitions. Nonetheless, the presence of outliers causes the cluster centres and their radii to be shifted, which leads to the identification of internal transitions among the surviving candidates. When outliers are present in a dataset, they can significantly affect the clustering results. Outliers are data points significantly different from the other points in the dataset and do not fit well into any existing clusters. As a result, when outliers are included in a cluster, the average distance between the members of that cluster increases. This increase in distance between the cluster members causes the cluster to become more diffuse, spreading out more widely than its ancestor clusters. As a result, the surviving clusters, after excluding outliers, may have a larger variance and less distinct boundaries, making it more difficult to separate them accurately from other clusters. This is particularly true if the dataset contains a high proportion of outliers.

### Generalized additive model

The Table 5 refers to the results of a GAM that includes a smoothing term for the variable $\texttt {outliers}$, and linear terms for $\texttt {separation}$ and $\texttt {covariance}$. The *p* value for $\texttt {covariance}$ is less than 5.10e−08, indicating strong evidence against the null hypothesis and is highly significant. Whereas, the *p* value for $\texttt {separation}$ is 0.998, which is greater than 0.05 and suggests that is not a statistically significant predictor of ${\texttt {hc}}$ function. This suggest that the separation between neighboring clusters does not affect the quality of clustering solutions in temporal datasets. However, the covariance structure between the variables significantly affect the clustering quality and its stability.

In the case of a smoothing term for the variable $\texttt {outliers}$, the *p* value is less than 0.0001 (represented as “$<2$e−16”), which indicates that the smoothing term for $\texttt {outliers}$ is highly statistically significant. This clearly indicates that the presence of a high percentage of $\texttt {outlier}$ increase the ${\texttt {hc}}$ function.

The ${\texttt {hc}}$ function is important in evolutionary clustering because it helps to ensure that the clustering algorithm is producing consistent and stable clustering solutions over time. A high ${\texttt {hc}}$ function is an indication that the updated clustering solution obtained as a result of new data is significantly different from the one generated initially. It is concluded that the presence of $\texttt {outliers}$ in the data stream resulted in low-quality clustering solutions that are highly unstable when new data points arrived at successive time points.

**Table 5:** The GAM model for ${\texttt {hc}}$ function as the response variable.

Parametric	Estimate	Std. Error	Pr(>|t|)
Intercept	0.536	0.053	2.45e−11 ***
cov	0.0587745	0.0083124	5.10e−08 ***
sep	− 0.0009883	0.3748288	0.998
	edf	Ref.df	*p* value
s(outliers)	1	1	$<2$e−16 ***

Signif. codes: 0 ‘***’ 0.001 ‘**’ 0.01 ‘*’ 0.05 ‘.’ 0.1 ‘ ’ 1

## Discussion

Monitoring changes in cluster solutions is an important aspect of analyzing temporal datasets. There are several algorithms proposed to monitor changes in cluster solutions of temporal datasets. Initially a clustering solution is generated from the dataset, which is then updated as new data becomes available to reflect the changes in the data. This can be done by re-running the clustering algorithm on the updated the solution. Consequently, the clusters undergo certain changes. As mentioned previously, the changes are categorized into external and internal transitions. The external transitions include candidates that survive, split, merge, emerge anew, or disappear, while the internal transitions consist of changes in location and cohesion. Stability analysis is a technique used to assess the robustness of clustering solutions to historic data. It involves assessing the stability and quality of the clustering solution using various techniques after updating it as new data becomes available. The stability of clustering solution at successive time points is carried out by examining the ${\texttt {hc}}$ function.

The ${\texttt {hc}}$ function in evolutionary clustering is a metric that is used to evaluate the performance of a clustering algorithm over multiple iterations. It is a measure of how well the clustering algorithm is able to maintain consistency and stability in the clustering solution over time. The ${\texttt {hc}}$ function works by comparing the clustering solutions from previous iterations of the algorithm to the current clustering solution. If the current solution is similar to the previous solutions, the ${\texttt {hc}}$ function will be low, indicating good performance. However, if the current solution is significantly different from the previous solutions, the ${\texttt {hc}}$ function will be high, indicating poor performance.

Findings of the study reveals that the presence of outliers in the stream does not affect the external transition of the clusters at successive time points. However, the surviving candidates are more spread out than the initial clusters. Similarly, low quality clustering solutions were obtained, i.e. having high ${\texttt {hc}}$, that were very inconsistent to the data of previous time point.

The clustering algorithm produced low-quality solutions with high ${\texttt {hc}}$ values that were not consistent with the data from the previous time point.

## Real-life data-set

The Melbourne House Prices dataset used in study was taken from Kaggle (Becker, 2021). This dataset comprises prices of properties sold in Melbourne between January 2016 and September 2017. The purpose of this data was to uncover the characteristics that impact property prices the most in the Melbourne Housing Market. This dataset has a total of 20 features (excluding the street address of houses) and 13580 observations. Houses with no price information have already been removed from the dataset. The dataset includes details on house selling prices, locations, and real estate brokerage firms. date of sale, year of construction, number of bathrooms, distance to city centre, size of the land, suburb, and the number of properties in the suburb are additional features.

The house prices were clustered at respective time points and changes in cluster solution were monitored. Detailed profiling of the clusters were carried out on the other attributes. The optimal number of clusters at respective time points was estimated using silhouette statistic. The stream was discritized on the selling date of the property.

**Fig. 2:**
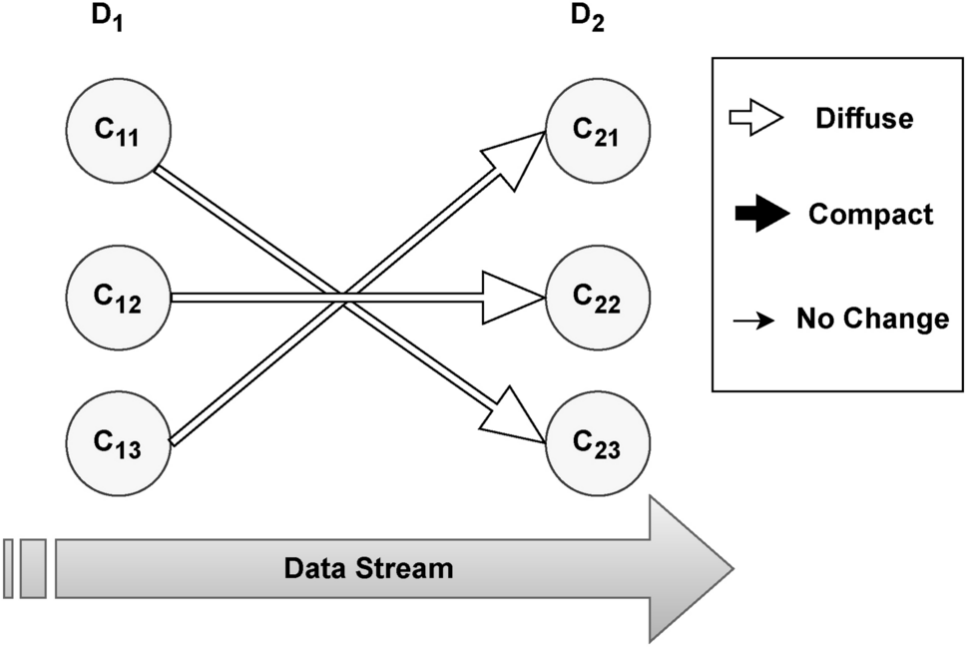
Cluster’s experiencing changes in Melbourne House Prices dataset containing outliers.

The Changes by the clusters at successive time points are shown in Fig. 2 below. The algorithm failed to identify any external transition, and all clusters survived i.e. cluster $C_{11}$ survived in $C_{23}$, cluster $C_{12}$ survived in $C_{22}$, and cluster $C_{13}$ survived in $C_{23}$. However, all surviving clusters underwent internal changes and spread out more than their ancestors.

**Fig. 3:**
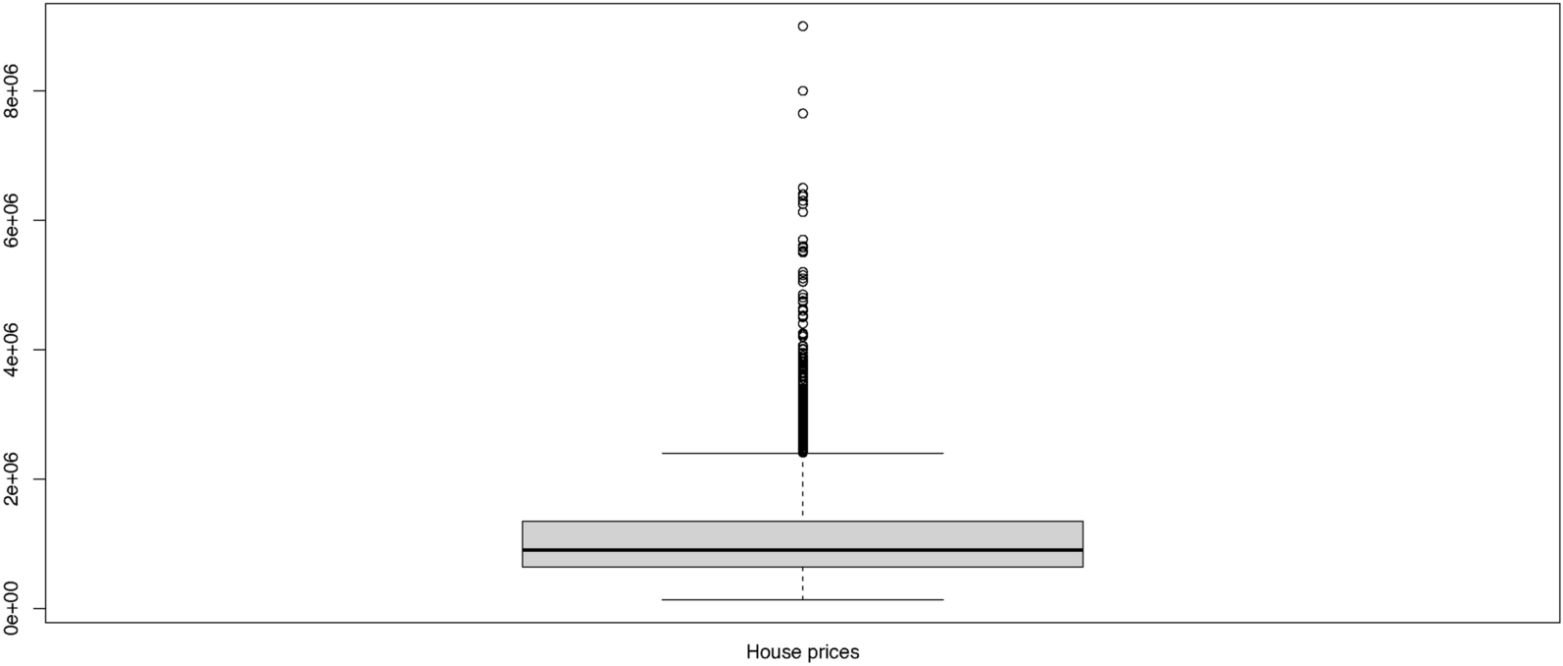
Box-plot of Melbourne House Prices dataset.

The *k*-Means method is affected by outliers since it seeks the average value among clusters. Figure 3 below shows the box-plot for house prices which indicates that the house prices include a huge number of outliers. These outliers might affect the cluster solutions and may affect the cluster transitions. These outliers are removed and then the cluster solutions are traced and monitored.

**Fig. 4:**
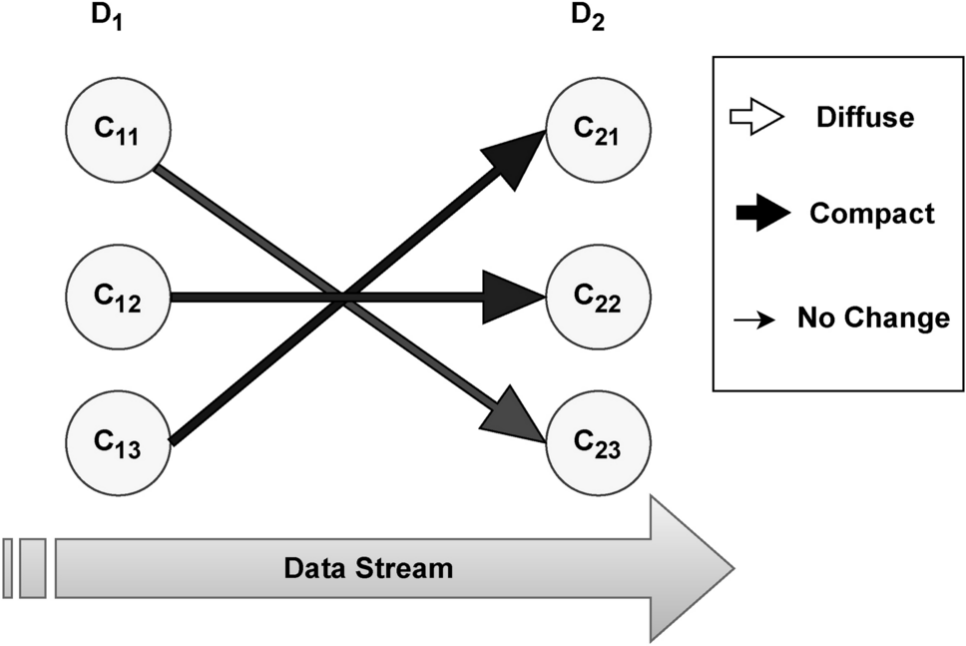
Cluster’s experiencing changes in Melbourne House Prices dataset after removing outliers.

Figure 4 below illustrates the changes in clustering solution of house prices after removing the outliers from the data-set. There were 21% outliers in the house price variable. All of the three clusters survived, with no internal transition detected.

Table 6 below demonstrates the cluster profiling of number of rooms, Cars, and Building are of the property before and after removing the outliers from the data-set. It is evident that the outliers affect the centroid and a shift in location was observed. However, if the outliers are removed from the data then there is no differenc in the centroids of the clsuters.

**Table 6:** Cluster profiling of variables related to the prices of property.

Variables	Outlier	$C_1$	$C_2$	$C_3$
Rooms	2016	3.1	2.5	3.4
	2017 (with outliers)	4.2	2.8	3.9
	2017 (without outliers)	3.1	2.6	3.4
Car	2016	2.2	1.3	1.6
	2017 (with outliers)	2.7	1.7	1.9
	2017 (without outliers)	2.3	1.7	1.5
Building Area	2016	293.4	109.4	173.7
	2017 (with outliers)	270.3	123.2	195
	2017 (without outliers)	291.6	112.2	170.9

All the synthetic streams were simulated at two time points

## Conclusion

Cluster analysis, a type of unsupervised learning, effectively uncovers patterns within a data stream by evaluating similarities among its data elements. Given that the entire stream isn’t accessible at once, many stream clustering methods adopt the windowing approach. This method involves segmenting the continuous data stream into discrete windows, allowing sequential processing of each window. New data continuously enters the stream while the oldest data exits, maintaining a fixed-size window that moves along the stream. This approach enables manageable chunk processing, aiding efficient analysis and clustering. Moreover, it conserves computational resources, making it feasible to handle large, continuous data streams in real-time. Subsequently, clustering solutions are generated at successive time points, and various algorithms have been developed to monitor and trace changes in these solutions over time, categorizing them as internal or external transitions. Although traditional batch clustering methods are known to be sensitive to outliers, the influence of outliers on stream clustering remains relatively unexplored. Consequently, there is a lack of comprehensive research and guidance on this subject. Hence, this study aims to address this gap by conducting a thorough analysis of how outliers impact the evolution of clusters in data streams.

Based on the findings of this study, it appears that outliers present in a data stream do not exert a substantial influence on the external transitions of clusters. However, their presence does have a detrimental effect on the internal transitions of the surviving clusters. Consequently, most clusters tend to become more dispersed compared to their predecessors. Even though the streams were simulated having same centroids, a noticeable shift in position of generated clusters is observed due to outliers. This underscores the significant impact of outliers on the overall clustering solution, despite their seemingly limited effect on the external transition of clusters.

## Assumptions and limitations

The study implements the MONIC framework, which assumes that every object must be classified into one and only one cluster. This assumption restricts the algorithm to only partitioning and hierarchical methods, as density-based algorithms assign some objects to outliers, which are not part of any cluster. If the dataset contains many outliers or noise, using only partitioning clustering algorithms may lead to less accurate results. Density-based clustering algorithms may be more effective with complex or irregular cluster shapes in the presence of outliers.

Similarly, the parameter selection of partitioning algorithms may introduce potential biases. For instance, in our study, we employed the same number of clusters at time points $t_1$ and $t_2$. This decision inherently limits the emergence of new clusters between these time points. As a consequence, the clustering solution may not fully capture the evolving structure of the data, potentially biasing our analysis towards a more static representation of the underlying phenomena. This limitation underscores the need for careful consideration of parameter settings and highlights the importance of exploring alternative approaches to accommodate the dynamic nature of streaming data.

Furthermore, we aimed to capture the complexity of the phenomenon as closely as possible, there are certain parameters that we may have overlooked. For instance, Atif et al.^30^ accounted for the impact of the number of variables on the evolution of clusters. However, dimensionality interacts with cluster sizes, leading to a complex relationship. Due to this complexity, we opted to focus solely on other factors and did not include dimensionality and cluster sizes in our analysis.

In future this study can be extended to the density-based algorithms to asses the impact of outliers on cluster evolution. Implementation of the density-based algorithms can effectively overcome these limitations. Dimensionality and cluster sizes will be studied in future investigations to further understand their interactions in presence of outliers.

## Data Availability

All data generated or analysed during this study are included in this published article.
