# Author Correction: Uncovering the impact of outliers on clusters’ evolution in temporal data-sets: an empirical analysis

**DOI:** 10.1038/s41598-025-94429-9

**Published:** 2025-03-20

**Authors:** Muhammad Atif, Muhammad Farooq, Muhammad Shafiq, Tmader Alballa, Somayah Abdualziz Alhabeeb, Hamiden Abd El-Wahed Khalifa

**Affiliations:** 1 Department of Statistics, University of Peshawar, Peshawar, Pakistan; 2 Institute of Numerical Sciences, Kohat University of Science and Technology, Kohat, Pakistan; 3 Department of Mathematics, College of Science, Princess Nourah Bint Abdulrahman University, P.O. Box84428, 11671 Riyadh, Saudi Arabia; 4 Department of Mathematics, College of Science, Qassim University, 51452 Buraydah, Saudi Arabia; 5 Department of Operations and Management Research, Faculty of Graduate Studies for Statistical Research, Cairo University, Giza, 12613 Egypt

Correction to: *Scientific Reports* 10.1038/s41598-024-75928-7, published online 28 December 2024

The original version of this Article contained an error in the spelling of the author Hamiden Abd El-Wahed Khalifa which was incorrectly given as Hamide Abd El-Wahed Khalifa.

The original Article has been corrected.

